# COVID-19 Myocarditis: Rationale for Early Diagnosis and Intervention

**DOI:** 10.7759/cureus.16494

**Published:** 2021-07-19

**Authors:** Syed S Fatmi, Rafaela Basso, Adnan Liaqat, Fateeha Tariq, Ramesh Swamiappan

**Affiliations:** 1 Internal Medicine, Southeast Health Medical Center, Dothan, USA; 2 Critical Care, Southeast Health Medical Center, Dothan, USA

**Keywords:** covid-19, myocarditis, echo cardiogram, covid-induced myocarditis, cardiac mri

## Abstract

Myocarditis is a common cardiovascular manifestation seen in patients diagnosed with severe acute respiratory syndrome coronavirus-2 (SARS-CoV-2) infection. However, because of the similarity of presentation with other cardiopulmonary pathologies, identification of coronavirus disease 2019 (COVID-19) related myocarditis can be challenging. Transthoracic echocardiography is a key component in initial diagnosis. COVID-19 related myocarditis is increasingly identified as an underlying problem in COVID-19 patients with low ejection fraction. Early recognition is critical with a low threshold for screening echocardiogram. Utilization of cardiac MRI (CMRI) can be helpful in recognition of early manifestations of COVID-19 myocarditis, with the added benefit of avoidance of invasive testing such as endomyocardial biopsy (EMB). Once diagnosis is established, disease-specific treatment can lead to rapid recovery of ventricular systolic function. We present a case series including two similar cases of COVID-19 myocarditis in which we utilized echocardiography as an early diagnostic tool and prompt treatment led to better prognosis.

## Introduction

The novel coronavirus disease 2019 (COVID-19) has been associated with significant morbidity and mortality primarily because of pulmonary involvement of this disease [1]. However, extra-pulmonary manifestations, particularly cardiovascular related, include acute myocardial infarction, arrhythmias, and myocarditis [2]. COVID-19 associated myocarditis is a diagnosis of exclusion and represents a diagnostic challenge, especially in subjects with a history of heart failure or other causes of cardiomyopathy. The incidence of COVID-19 myocarditis is increasing, and studies have attributed as much as 7% of COVID-19 cases to myocarditis [3]. Clinical presentation of COVID-19 myocarditis varies significantly due to the overlap of its symptoms with other disease processes such as pulmonary disease and right-sided heart failure. COVID-19 myocarditis can present with fulminant heart failure with or without typical pulmonary manifestations. Fulminant heart failure with troponin elevation can mimic other cardiac conditions, such as acute coronary syndrome, Takatsubo cardiomyopathy, and ischemic cardiomyopathy, and this makes early detection of COVID-19 myocarditis a challenge [4,5]. Although the exact pathophysiology of COVID-19 myocarditis is not well known, it is believed to be from direct damage to cardiomyocytes by COVID-19 virus through binding to angiotensin-converting enzyme 2 (ACE2) receptors resulting in cytokine release syndrome (CRS) which is mediated by dysregulated response from type I and type II helper T-cells. Initiation of this response results in injury to cardimyocytes and over activation of an immune system with possible interferon mediated hyper-activation of the immune system [3]. The resulting myocarditis can result in focal or global myocardial inflammation and necrosis, eventually resulting in ventricular dysfunction [6]. Early recognition and prompt treatment of myocarditis in patients with COVID-19 infection is crucial. There is limited data regarding long-term sequelae, or prognosis related to COVID-19 myocarditis but as described in the two cases below, early detection and management have shown improved outcomes. 

## Case presentation

Case # 1

**Figure 1:**
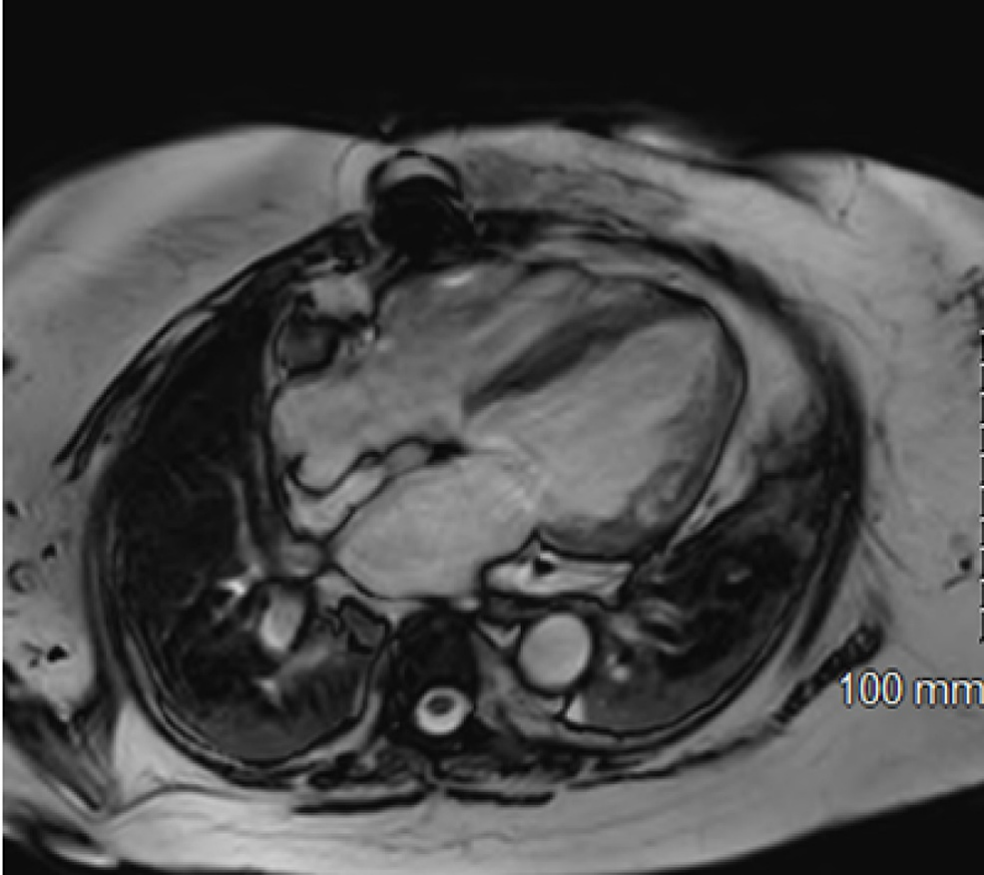
Cardiac MRI Combination of transmural and subendocardial enhancement involving the septal wall extending into the inferior septal region

A 69-year-old Caucasian female with a past medical history of coronary artery disease, status post percutaneous coronary intervention, hypertension, diabetes mellitus type 2, and paroxysmal atrial fibrillation, presented to our emergency department with substernal chest pain with worsening shortness of breath. On physical exam, the patient was barely responsive, tachycardia, and hypotensive. She had significant hypoxia and subsequently was intubated and requiring mechanical ventilation. Initial diagnostic work up revealed ST-segment elevation in precordial leads on electrocardiogram, Troponin 0.56 and X-ray chest findings were consistent with bilateral pulmonary infiltrates. Further testing showed positivity for COVID-19. We immediately transferred the patient to the cardiac catheterization unit due to concern for possible myocardial infarction. No acute coronary artery occlusion was identified but the patient was found to have significantly reduced left ventricular ejection fraction (LVEF). Her LVEF was 20%, less than her recent baseline of 45%. She remained critically ill and on mechanical ventilation. Because of concerns for cardiogenic shock secondary to COVID-19 myocarditis and keeping in view negative cardiac catheterization, cardiac magnetic resonance imaging (CMRI) was planned. CMRI showed sub-endocardial/transmural enhancement occupying 50% of myocardial thickness (Figure 1). CMRI findings were consistent with suspicion of COVID-19 related myocarditis. The patient was given optimal congestive heart failure regimen and continued to show improvement. She was eventually discharged home after a complicated course requiring mechanical ventilation. Transthoracic echocardiogram approximately two months after discharge showed LVEF of 40%.

Case # 2

**Figure 2:**
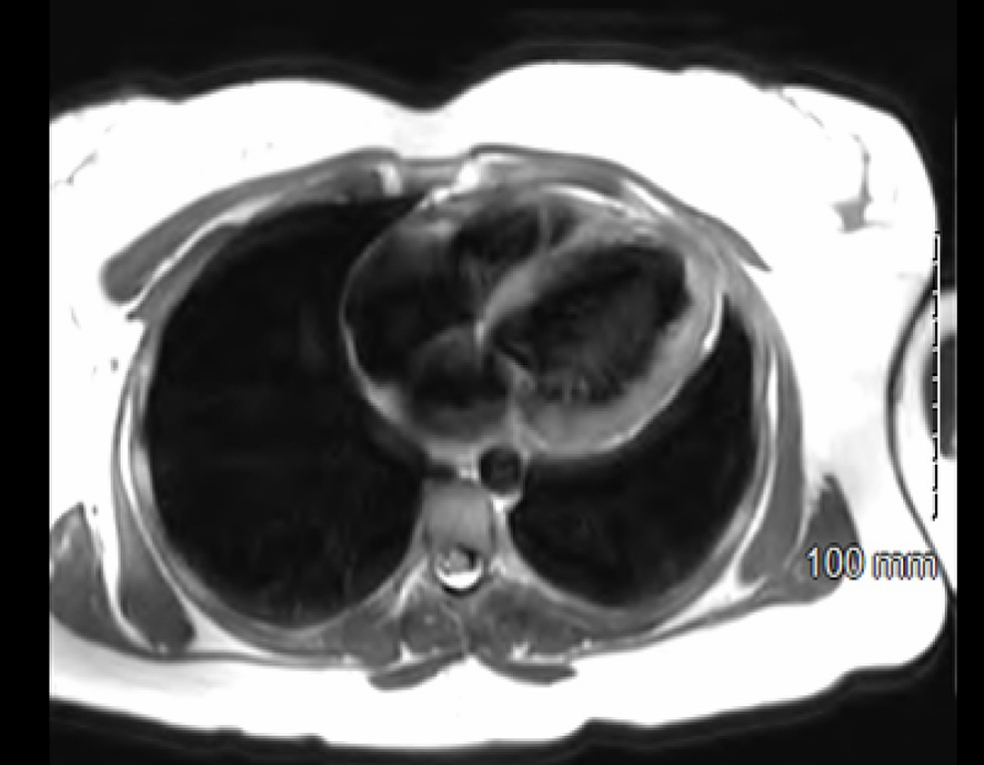
Cardiac MRI Cardiac MRI showed findings of epicardial enhancement and edema compatible with apical anterior lateral wall myocarditis

**Figure 3:**
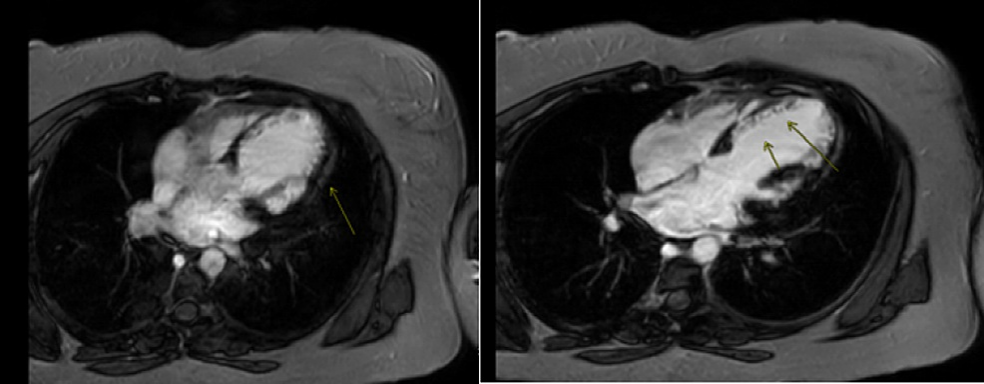
Cardiac MRI Endocardial enhancement along the apical anteroseptal wall with edema in keeping with acute infarction, this is greater than 50% thickness of the septum

A 41-year-old female with a past medical history of ablation procedure secondary to frequent premature ventricular contractions, recent diagnosis of COVID 19 infection without hospitalization presented to the cardiology clinic with intermittent chest pain and shortness of breath. On physical exam, she was vitally stable and no significant findings were noted. Initial electrocardiogram was consistent with normal findings and troponins were negative. Transthoracic echocardiogram revealed akinesis of anteroseptal walls consistent with an infarct and dilated left ventricle with an estimated LVEF 45%. These findings warrant further diagnostic investigations. Lexiscan stress test showed perfusion defect concerning for possible infarct with LVEF by single-photon emission computed tomography (SPECT) scan calculated to be at 29%. Repeat transthoracic echocardiography estimated LVEF 30%. Patient underwent cardiac catheterization. Cardiac catheterization findings were consistent with dilated non-ischemic cardiomyopathy with LVEF 30%. CMRI was planned to further investigate the underlying cause. CMRI showed epicardial enhancement and edema compatible with apical anterior and anterior lateral wall myocarditis with LVEF approximately 28% (Figures 2-3). These findings raised the suspicion of COVID-19 associated myocarditis. Patient was started on appropriate medication for congestive heart failure. A multigated acquisition (MUGA) scan, approximately four months after discharge indicated marked improvement LVEF to 47% with complete resolution of her symptoms. 

## Discussion

**Figure 4:**
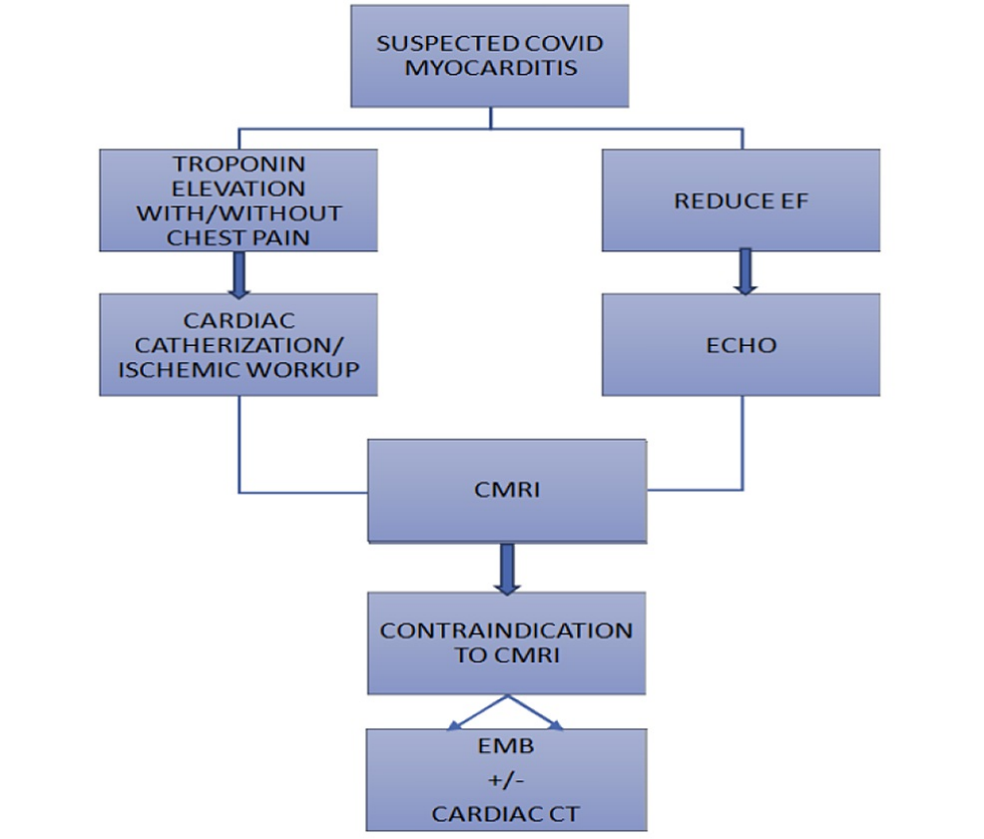
Diagnostic Approach to COVID-19 Myocarditis CMRI: cardiac magnetic resonance imaging; EF: ejection fraction; EMB: endomyocardial biopsy; COVID-19: coronavirus disease 2019

COVID-19 myocarditis is usually a diagnosis of exclusion and typically found as an incidental finding on transthoracic echocardiography. Various studies have described cases of COVID-19 myocarditis, but early recognition of myocarditis in patients with COVID-19 infection remains critical for prompt recovery. As myocarditis symptoms overlap with respiratory symptoms, we can consider the use of transthoracic echocardiography as a screening tool in patients with acute respiratory failure or worsening symptoms related to COVID-19. Bedside transthoracic echocardiography is usually performed in critical care unit patients with a history of ischemic cardiomyopathy or to gauge right heart pressures in the setting of respiratory failure and cardiogenic shock. Keeping a low threshold to get transthoracic echocardiography in COVID-19 patients might be of benefit in the early detection of COVID-19 myocarditis and can result in improved outcomes. Although some pathognomonic findings exist, some signs of myocarditis on transthoracic echocardiography are nonspecific to COVID-19 myocarditis which includes increased wall thickness, chamber dilation, and possible pericardial effusion with worsening systolic function [3,7]. However, a definitive diagnosis of COVID-19 myocarditis can be established with endomyocardial biopsy (EMB) but it is rarely employed due to being invasive. CMRI should be considered as an alternative. The diagnostic approach to COVID-19 myocarditis is shown in Figure 4.

Imaging and early utilization of CMRI

Imaging findings of COVID-19 related myocarditis on CMRI include; myocardial dysfunction including impaired LVEF, systolic strain, pericardial enhancement with or without effusion, diffuse myocardial edema seen on T1 and T2, late gadolinium enhancement, and signs of stress induced cardiomyopathy with subendocardial edema and enhancement [8]. CMRI findings for COVID-19 are recognized by revised Lake Louise consensus criteria [9,10]. Pericardial effusion has been seen in upto 57% of the patients with myocarditis and it can be a possible contributing factor in cardiogenic shock in such patients [10]. We think CMRI is an underutilized modality, that can differentiate the etiology of systolic dysfunction between COVID-19 and other related underlying conditions with similar presentation. CMRI can play a vital role in early recognition and diagnosis of COVID-19 myocarditis. However, there are some limitations to the use of CMRI including low hemodynamic status, cost of tests, medical contraindications to magnetic resonance imaging (such as non-compatible pacemakers or aneurysm clips). In cases with any factor limiting the use of CMRI, EMB and contrast-enhanced computed tomography (CECT) can be useful option.

## Conclusions

In patients with COVID-19 myocarditis, early detection and prompt treatment can lead to improved outcomes. COVID-19 myocarditis presentation can mimic worsening respiratory function because of COVID-19 and initial diagnostic workup can raise suspicion of acute coronary syndrome, as in our cases elevated markers of myocyte injury (troponins), and worsening LVEF were found. We can consider transthoracic echocardiography as an early screening tool as it is minimally invasive, but it has its own limitations, especially for determining the underlying etiology. CMRI can provide us with more details and can be a diagnostic modality for COVID-19 myocarditis. EMB and CECT can also be helpful in cases with contraindications to CMRI use. 
